# Raman Spectroscopic Study of Five Typical Plasticizers Based on DFT and HF Theoretical Calculation

**DOI:** 10.3390/foods12152888

**Published:** 2023-07-29

**Authors:** Tong Sun, Yitao Wang, Mingyue Li, Dong Hu

**Affiliations:** College of Optical, Mechanical and Electrical Engineering, Zhejiang A&F University, Hangzhou 311300, China; 2020604021041@stu.zafu.edu.cn (Y.W.); lmy@stu.zafu.edu.cn (M.L.); 20180047@zafu.edu.cn (D.H.)

**Keywords:** PAEs, Raman, DFT, HF, theoretical study

## Abstract

Phthalic acid esters (PAEs) are the most commonly used plasticizers, and long-term or high levels of exposure to PAEs have a huge potential risk to human health. In this study, the theories of Hartree–Fock (HF) and density functional theory (DFT) with different hybrid methods and basis sets were used to calculate the theoretical Raman spectra of five PAEs, and the comparison of calculated spectra between different theories, hybrid methods, and basis sets was conducted to determine the suitable theory with hybrid method and basis set for PAEs. Also, the Raman vibrations were assigned to the Raman peaks of PAEs according to the theoretical and experimental Raman spectra. The results indicate that DFT is more suitable for the theoretical study of PAEs than HF. In DFT, the hybrid method of B3LYP is more applicable to the theoretical study of PAEs than B3PW91, and the basis set of 6-311G(d, p) obtains the most consistent theoretical Raman spectra with the experimental spectra for PAEs. This study finds the optimal combination of the theoretical method and basis set for PAEs, and it will contribute to the establishment of the Raman fingerprint and the development of rapid detection for PAEs in the future.

## 1. Introduction

Plasticizers are polymer additives that are commonly used in packaging materials to increase plasticity [1]. Phthalic acid esters (PAEs) are the most commonly used plasticizers, which can enter the body with people’s breath, diet, and even skin contact. When PAEs accumulate to a certain extent in the body for a long time, they can be harmful to human health, and will cause feminization of men, increase the risk of breast cancer in women, and leads to deformity and cancer [2]. In recent years, the frequent occurrence of excessive PAEs in food has attracted widespread concern and great importance from the government and society. In 2011, Taiwan’s Food and Drug Administration found high concentrations of Di(2-ethyl)hexyl phthalate in a batch of probiotic ingredients [3]. Since then, PAEs have become known to the public. In 2012, the Plasticizer incident of Chinese Baijiu caused a sensation [4]. The incidents of artificially added PAEs in milk tea in 2017 and excessive PAEs in Ladue Blue Joe walnut oil in 2019 are even more controversial [5]. Therefore, it is very necessary to effectively monitor PAEs in food to protect people’s health.

In recent years, Raman spectroscopy has been widely used in the field of food safety [6,7,8] and gradually applied to the detection of PAEs due to its characteristics of no sample pretreatment, fast detection speed, and response to molecular fingerprint information. Wu et al. [9] prepared homogeneous AuNPs films for the detection of Di (2-ethyl)hexyl phthalate in sorghum wine. Zhou et al. [10] formed Ag@Fe3O4@PEI nanoparticles, then modified them with cyclodextrin (β-CD). Finally, 1.3 mg/kg of BBP in white wine was able to detect using this substrate. Cao et al. [11] prepared Au-Ag-S nanostructured substrates using a one-pot method and used them for the detection of Di(2-ethyl)hexyl phthalate in juice. Wang et al. [12] and Wu et al. [13] used 2D silver plate and AuNPs as enhanced substrates to detect PAEs in edible oils. However, few of these studies incorporate structural theory, whereas identifying PAEs requires the incorporation of structural theory. Structure theory includes the semi-empirical method, density functional theory (DFT), and ab Initio [14,15]. It can help to understand the experimental results [16,17,18]. Ji et al. [19] assigned the Raman vibrations to the eight Raman peaks of the Dimethyl phthalate by DFT 6-31+G(d) calculations. Liu et al. [20] simulated the theoretical Raman spectra of di(2-ethyl)hexyl phthalate, dibutyl phthalate, and diethyl phthalate using DFT 3-21G; then, the theoretical Raman spectra with the corresponding experimental Raman spectra were compared and analyzed. Qiu et al. [21] calculated the Raman spectra of dimethyl phthalate, dibutyl phthalate, Di-n-octyl phthalate and their derivatives in the gaseous environment using DFT B3LYP 6-31g(d), which contributed to the studies of PAEs. Xu et al. [22] used DFT 6-31G(d) to calculate the theoretical spectra of di(2-ethyl)hexyl phthalate, dibutyl phthalate, and butyl benzyl phthalate. The theoretical Raman spectra were consistent with the experimental spectra, and the Raman vibrations were assigned to Raman peaks. Zuo et al. [23] used molecular dynamics simulations and DFT to reveal inter-molecular interactions of phthalic acid esters. 

There is no comparison of theoretical Raman spectra of PAEs calculated by different theoretical methods and basis sets in the existing studies. The explanation of basis sets can be found in the second paragraph of Section 2.3. The theoretical Raman spectra calculated by some theoretical methods will have many spurious peaks, which are not found in the experimental Raman spectra; this will interfere with the analysis of the experimental data and cause errors. In addition, existing studies have only assigned the Raman vibrations to individual Raman peaks, but not to all major Raman peaks. In this research, the theoretical Raman spectra of five typical PAEs were simulated by different theoretical methods and basis sets, and were compared with the experimental Raman spectra in order to obtain the most applicable theoretical method and basis set for PAEs, which can effectively reduce the influence of spurious peaks on the analysis of PAEs detection in Food or other products. Also, all Raman peaks of the five PAEs were assigned according to the theoretical and experimental Raman spectra.

## 2. Materials and Methods

### 2.1. Materials and Equipment

Dimethyl phthalate (DMP), diethyl phthalate (DEP), and dibutyl phthalate (DBP) reagents were purchased from Sinopharm Chemical Reagent Co. (Shanghai, China) Di(2-ethyl)hexyl phthalate (DEHP) and diisononyl phthalate (DINP) reagents were purchased from Aladdin Reagent Co. (Shanghai, China) DMP, DEP, DBP, DEHP, and DINP are all analytical pure reagents with a purity greater than 99.5%. Table 1 shows the details of the five typical PAEs.

A handheld portable Raman spectrometer (Bruker, Germany) equipped with dual wavelength lasers of 785 nm and 852 nm was used in this study. The spectral range, spot diameter, and spectral resolution of the spectrometer are 3200~300 cm^−1^, 1~2 mm, and 10~12 cm^−1^, respectively.

### 2.2. Spectral Acquisition

**Figure 1:**
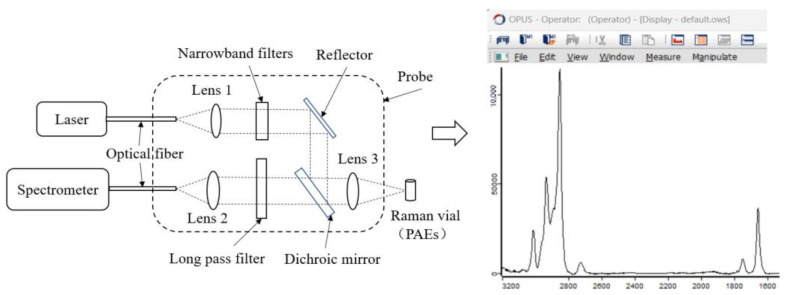
Schematic diagram of Raman spectrum acquisition. PAEs: phthalic acid esters.

In order to reduce the influence of other impurities on the experimental Raman spectra of five PAEs, the analytical pure PAE samples were used for collecting Raman spectra. For DMP spectral acquisition, 3 mL of analytical pure DMP sample was put in the Raman vial first, then the Raman vial was placed into the liquid measurement accessory of the Raman instrument. After that, the DMP sample is irradiated with laser wavelengths of 785 nm and 852 nm in sequence, and the generated Raman spectrum signal passes through a lens, dichroic mirror, and long pass filter, and is then detected by the Raman spectrometer to obtain DMP Raman spectra at two laser wavelengths. Due to the fact that the positions of the fluorescence peaks do not change with the incident laser wavelength, while the positions of the Raman peaks will change with the incident laser wavelength, matching the Raman spectra of DMP at two incident laser wavelengths through software of OPUS8.7.31 can effectively eliminate fluorescence interference. Finally, the Raman spectrum of DMP that had eliminated fluorescence signal was obtained. The Raman spectra of DEP, DBP, DEHP, and DINP were acquired in the same way. The integration time and number of scans were both set to 6 s and 3, respectively, for five PAEs. Figure 1 shows the schematic diagram of Raman spectrum acquisition.

### 2.3. Theoretical Calculation

The Hartree–Fock method (HF) is one of the ab initio methods, which is based on the Schrodinger equation [24]. DFT is a method for studying the electronic structure of multi-electron systems, which has a wide range of applications in the study of the properties of molecules and condensed matter. It is one of the most commonly used methods in the field of computational materials science and computational chemistry in condensed matter physics [25,26,27]. There are many hybrid methods in DFT. The hybrid methods of B3LYP (Becke-3 exchange with Lee–Yang–Parr gradient-corrected correlation functional) and B3PW91 (B3 exchange + PW91 correlation) are the most used in the calculation of organic matter [28]. Both B3LYP and B3PW91 are exchange-correlated general functions with similar calculated results [29], but the specific results are related to the studied substances.

The basis set is the second component of the theoretical calculation, and using a basis set means selecting a region of space where each electron is located [30]. For example, 6-311G+(2d, p): the first 6 refers to the six Gaussian functions describing the inner layer electrons; the latter 311 means that each valence orbit is represented by three basis functions, which are fitted by 3, 1, and 1 original functions, respectively; G means Gaussian basis set; d means one additional polarization function for each heavy atom (non-hydrogen atom); p means one additional polarization function for the hydrogen atom adds a polarization function; + means to add a dispersion function to the heavy atom; if the hydrogen atom also wants to add a dispersion function, then + is replaced by ++ [31]. The larger the basis set, the fewer the constraints imposed on the electrons and the more accurate the approximation of the true molecular wave function. The choice of the basis set depends on different accuracy requirements, theoretical approaches, and research object systems, etc. [32,33,34].

In this study, structural models of five PAEs(DMP, DEP, DBP, DEHP, and DINP) were constructed. Then, the two theories of DFT and HF with 6-31G(d) were used to calculate the theoretical Raman spectra of five PAEs, and the spectra of five PAEs calculated by DFT and HF were compared in order to obtain the suitable theory for PAEs. In DFT theory, the hybrid methods of B3LYP and B3WP91 were chosen, and the spectra calculated by B3LYP and B3WP91 of DFT were compared to determine which specific method would be more suitable. After that, different basis sets, (3-21G, 6-31G(d), 6-311G(d, p), and 6-311G+(d, p), were used to simulate theoretical spectra of five PAEs, and the results were compared to choose the most applicable basis set for PAEs. Finally, the most applicable theoretical Raman spectra combined with experimental Raman spectra were analyzed to assign the Raman vibrations to the Raman peaks. All the theoretical calculations are prepared using the Gaussian09 (version 9.5), software.

## 3. Results

### 3.1. Molecular Structure of PAEs 

**Figure 2:**
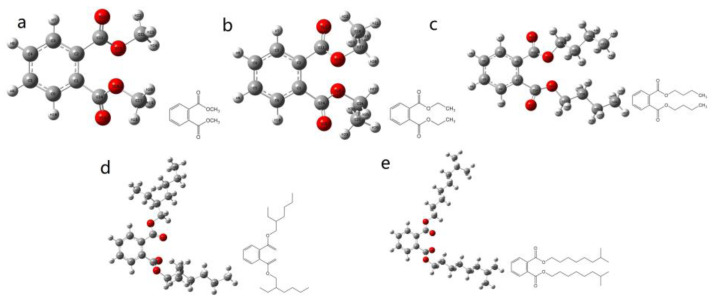
Optimized molecular structure diagram of PAEs and its molecular formula. (**a**): DMP; (**b**): DEP; (**c**): DBP; (**d**): DEHP; (**e**): DINP. DMP: dimethyl phthalate; DEP: diethyl phthalate; DBP: dibutyl phthalate; DEHP: di(2-ethyl)hexyl phthalate; DINP: diisononyl phthalate.

The structural models of the five PAEs (DMP, DEP, DBP, DEHP, and DINP) and their molecular formulae are shown in Figure 2a–e. It can be found that the structure of DMP consists of a benzene ring, two carboxyl groups, and two methyl groups, and the structure of DMP is the simplest among these PAEs. The study of DMP has important reference values for other PAEs [35]. Also, the other four PAEs are relatively typical structures, which are important for the study of PAEs [36].

### 3.2. Experimental Raman Spectra of PAEs

**Figure 3:**
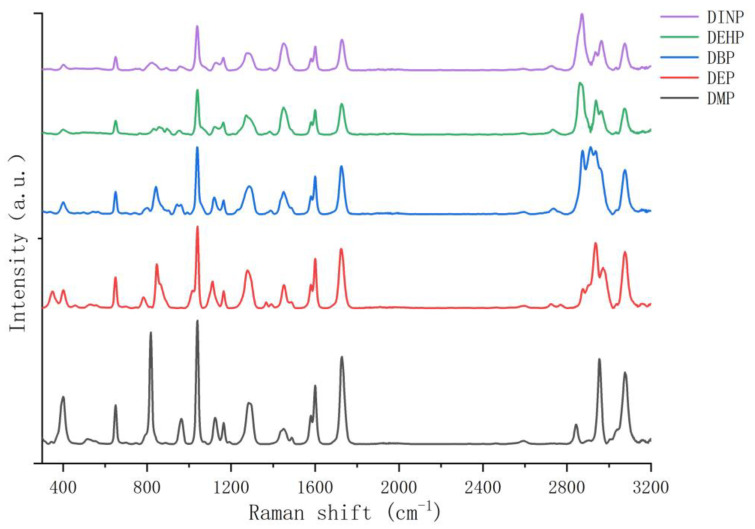
Experimental Raman spectra of phthalic acid esters. DINP: diisononyl phthalate; DEHP: di(2-ethyl)hexyl phthalate; DBP: dibutyl phthalate; DEP: diethyl phthalate; DMP: dimethyl phthalate.

Figure 3 shows the experimental Raman spectra of five PAEs. From Figure 3, it can be seen that the Raman peaks in the range of 2800~3200 cm^−1^ are very heterogeneous, and the peaks in this interval overlap with those of many solvents such as ethanol. Therefore, the range of 300~2000 cm^−1^ is chosen for this study. From Figure 3, it can be seen that the common Raman peaks of the five PAEs are 400, 650, 1040, 1120, 1160 1284, 1450, 1580, 1600, and 1726 cm^−1^. The unique Raman peaks of DMP are 818 and 964 cm^−1^; the unique Raman peaks of DEP are 352, 784, and 848 cm^−1^; the unique Raman peaks of DBP are 810, 842, 940, and 962 cm^−1^; the unique Raman peaks of DEHP are 834, 858, 894, and 956 cm^−1^; and the unique Raman peaks of DINP are 822, 900, and 960 cm^−1^. The partial experimental Raman peaks of PAEs in this study are consistent with the peaks of 650, 1040, 1580, 1600, and 1726 cm^−1^ for DEHP and DBP in the literature [22]. They are basically consistent with the peaks of 1038, 1120, 1578, 1599, and 1723 cm^−1^ for DEHP, DEP, and DBP in the literature [20], and the peaks of 403, 653, 1043, 1127, 1167, 1585, 1605, and 1731 cm^−1^ for eight PAEs in the literature [37].

### 3.3. Comparison of HF and DFT Methods

Different methods have different calculation accuracy for different substances, and the accuracy of the Raman shifts calculated by HF and DFT needs to be verified by comparison with experimental data. From the literature [20,21,22], it is clear that theoretical studies have been carried out on PAEs, but only one or two methods are selected for study, and no comparative studies have been conducted. The Raman spectra calculated by some theoretical methods will have many spurious peaks that are not present in the experimental spectra, which will cause interference and errors in the analysis of experimental data; so, it needs to find the suitable theoretical method for PAEs.

**Figure 4:**
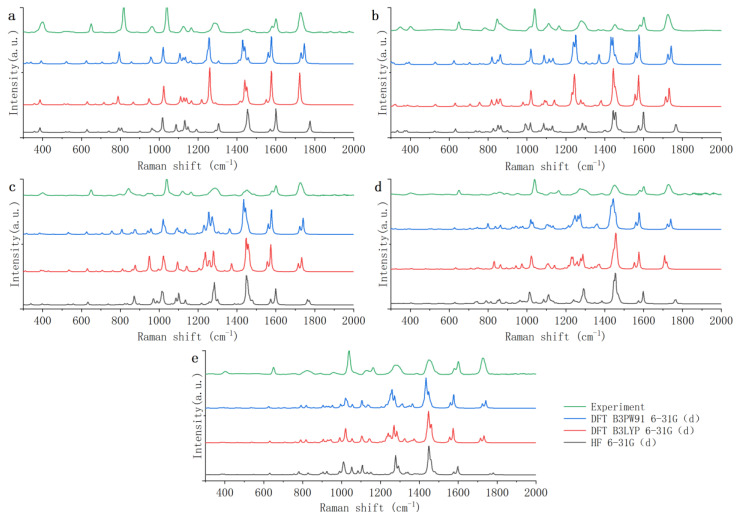
Theoretical Raman spectra of five phthalic acid esters based on HF and DFT: (**a**) DMP; (**b**) DEP; (**c**) DBP; (**d**) DEHP; (**e**) DINP. HF: Hartree–Fock method; DFT: density functional theory; DMP: dimethyl phthalate; DEP: diethyl phthalate; DBP: dibutyl phthalate; DEHP: di(2-ethyl)hexyl phthalate; DINP: diisononyl phthalate.

Figure 4 shows the theoretical Raman spectra of the five PAEs (DMP, DEP, DBP, DEHP, and DINP) calculated by HF and DFT with the 6-31G(d) basis set. Because many theoretical spectra have some offset errors from experimental spectra, it is necessary to use the scale factors from the database of frequency scale factors for electronic model chemistries [38] to correct the theoretical spectra in order to eliminate the offset error to the greatest extent [39,40]. From the scale factors database, it can be seen that the scale factors of HF 6-31G(d), DFT B3LYP 6-31G(d), and DFT B3PW91 6-31G(d) are 0.885, 0.952, and 0.947, respectively. In this study, the theoretical spectra are the spectra after correction.

As shown in Figure 4, the theoretical Raman spectra calculated by HF and DFT show good agreement as a whole with the experimental Raman spectra, but there are large differences in individual Raman peaks. Comparing the theoretical Raman spectra calculated by HF with the experimental Raman spectra, it is found that the peaks of the five PAEs have common differences. The wide peaks of 1284 and 1450 cm^−1^ all become sharp, and the strong peaks of 1726 cm^−1^ become much weaker. The theoretical Raman peaks all have a red shift in the band of 300~800 cm^−1^, while all have a blue shift in the band of 1500~2000 cm^−1^. In addition to the above common differences, the theoretical Raman spectra of the five PAEs also individually have a lot of spurious Raman peaks. Among them, the theoretical Raman spectra of DMP, DEP, and DBP have more spurious peaks. In terms of DMP, the peak of 818 cm^−1^ is divided into peaks of 790 and 806 cm^−1^, the peak of 1120 cm^−1^ is divided into peaks of 1130 and 1146 cm^−1^, and there is a spurious peak of 1080 cm^−1^. In terms of DEP, the peak of 1120 cm^−1^ is divided into peaks of 1086, 1102, and 1126 cm^−1^, and there is a spurious peak of 992 cm^−1^. In terms of DBP, the peaks of 940 and 962 cm^−1^ are shifted to 970 and 990 cm^−1^, and the peak of 1120 cm^−1^ is divided into peaks of 1086 and 1110 cm^−1^. Therefore, it can be seen that the theoretical Raman spectra calculated by HF 6-31G(d) have so many errors. This may be because HF ignores most of the electronic correlations [41], which makes the theoretical spectra of PAEs inaccurate.

There are also some errors in the theoretical Raman spectra calculated by DFT. However, the theoretical Raman spectra calculated by B3LYP and B3PW91 of DFT both have fewer spurious peaks than the theoretical Raman spectra calculated by HF. Therefore, DFT is more applicable to theoretical studies of PAEs. Then, the theoretical Raman spectra calculated by B3LYP and B3PW91 are further compared. It is found that the theoretical Raman spectra calculated by the B3PW91 have slightly more spurious peaks. In terms of DMP, there are two spurious peaks of 320 and 330 cm^−1^. In terms of DEHP, there is a spurious peak of 800 cm^−1^. Peak of 1726 cm^−1^ in five PAEs are all divided into two Raman peaks. In addition, the theoretical calculation time of the two methods is not significantly different. So, it can be concluded that the DFT B3LYP method is more applicable to the theoretical study of PAEs.

### 3.4. Different Basis Sets with DFT B3LYP

The above results show that the DFT B3LYP method is more suitable for the study of PAEs. Different basis sets of DFT B3LYP have different calculation accuracy, and further theoretical studies are needed to select the suitable basis set. 

**Figure 5:**
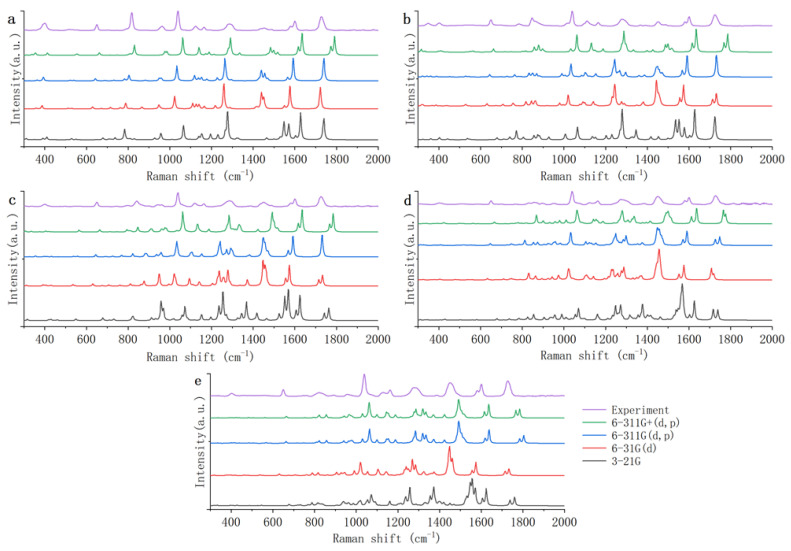
Theoretical Raman spectra of five phthalic acid esters calculated by DFT B3LYP with different basis sets: (**a**) DMP; (**b**) DEP; (**c**) DBP; (**d**) DEHP; (**e**) DINP. DFT: density functional theory; DMP: dimethyl phthalate; DEP: diethyl phthalate; DBP: dibutyl phthalate; DEHP: di(2-ethyl)hexyl phthalate; DINP: diisononyl phthalate.

Figure 5 shows the theoretical Raman spectra of five PAEs (DMP, DEP, DBP, DEHP, and DINP) calculated by DFT B3LYP, with 3-21G, 6-31G(d), 6-311G(d, p), and 6-311G+(d, p) basis sets. From the scale factors database, it can be seen that the scale factors of DFT B3LYP 6-311G(d, p) is 0.9708. The scale factors of DFT B3LYP 6-31G(d) is as above, and the scale factors of 3-21G and 6-311G+(d, p) are not found in the scale factors database.

As shown in Figure 5, compared with the other three basis sets, the theoretical Raman spectra of the 3-21G basis set have significantly more spurious peaks. Comparing the theoretical Raman spectra calculated by the 3-21G basis set with the experimental Raman spectra, it is found that the peaks of the five PAEs have common differences. The peaks of 650 cm^−1^ are all shifted to 680 cm^−1^; the peaks of 1120 cm^−1^ are all divided into peaks of 1138 and 1156 cm^−1^; the peaks of 1160 cm^−1^ are all shifted to 1190 cm^−1^; and the wide peaks of 1450 cm^−1^ are all divided into peaks of 1548 and 1570 cm^−1^. In addition to the above common differences, the theoretical Raman spectra of the five PAEs also individually have many spurious Raman peaks. This may be because the 3-21G basis set has only three original functions fitting per nuclear orbital basis function [42], which makes the theoretical spectra of PAEs inaccurate. Therefore, the 3-21G basis set is not applicable to the theoretical study of PAEs.

The theoretical Raman spectra calculated by the 6-31G(d) basis set have slightly more spurious peaks than the theoretical Raman spectra calculated by the 6-311G(d, p) and 6-311G+(d, p) basis sets. Comparing the theoretical Raman spectra calculated by 6-31G(d) with the experimental Raman spectra, it is found that the peaks of the five PAEs have common differences. The wide peaks of 1450 cm^−1^ all turn into a sharp peak and the intensity increases too much. The peaks of 1726 cm^−1^ are all divided into peaks of 1706 and 1720 cm^−1^. In addition to the above common differences, the theoretical Raman spectra of the five PAEs also have some spurious peaks. It may be because the 6-31G(d) basis set is represented by two basis functions per valence orbit, which is one function less than the other two basis groups; so, the accuracy of the 6-31G(d) basis set is a bit worse for PAEs.

The difference between the theoretical spectra calculated by 6-311G(d, p) and 6-311G+(d, p) basis sets is extremely small, and theoretical spectra are both in good agreement with the experimental spectra. However, the scale factors of the 6-311G+(d, p) basis set is not found in the scale factors database. Therefore, compared with the Raman peaks of the experimental spectra, the Raman peaks of the theoretical spectra calculated by the 6-311G+(d, p) basis set are all blue shifted as a whole. In addition, because the 6-311G+(d, p) basis set has more plus dispersion functions on heavy atoms than 6-311G(d, p), the calculation of 6-311G+(d, p) takes nearly three times longer time than 6-311G(d, p). Therefore 6-311G(d, p) is more appropriate for the theoretical study of PAEs.

In summary, the DFT B3LYP 6-311G(d, p) is most suitable for the theoretical study of PAEs. However, the theoretical spectra calculated by DFT B3LYP 6-311G(d, p) still have some differences with experimental Raman spectra in some details. Therefore, the theoretical Raman spectra obtained by DFT B3LYP 6-311G(d, p) were further analyzed. Table 2 shows the common Raman peaks in the theoretical and experimental Raman spectra of the five PAEs. There are some differentiated peaks in theoretical Raman spectra. Compared with the results calculated using the DFT B3LYP 6-311G (d, p) method in the literature, the results of DEHP in this study are basically consistent with the results of DEHP in the literature [37]. In the literature, the experimental and theoretical Raman peaks of DEHP are 399, 653, 1043, 1127, 1167, 1585, 1605, 1731, and 385, 645, 1043, 1134, 1163, 1583, 1608, 1742, 1751 cm^−1^, respectively. 

In addition to the peaks in the Table 2, there are still a few other peaks in the Raman spectra of PAEs. Compared with the experimental spectrum, the theoretical spectrum of DMP has two more peaks of 782 and 948 cm^−1^, which are extremely weak and negligible. The theoretical spectrum of DEP has four more Raman peaks of 342, 834, 870, and 990 cm^−1^ than the experimental spectrum. The peak of 342 cm^−1^ can be regarded as the differentiated peak from the peak of 360 cm^−1^, peaks of 834 and 870 cm^−1^ can be regarded as the differentiated peaks from the peak of 850 cm^−1^, and peak of 990 cm^−1^ can be ignored because its peak strength is small. The theoretical spectrum of DEHP has two more Raman peaks of 924 and 982 cm^−1^ than the experimental spectrum, and peaks of 924 and 982 cm^−1^ can be regarded as the differentiated peaks from the peak of 958 cm^−1^. The theoretical spectrum of DINP has one more Raman peak of 858 cm^−1^ than the experimental spectrum, which can be regarded as the differentiated peak from the peak of 822 cm^−1^. The peaks of the theoretical spectrum of DINP are blue-shifted by nearly 40 cm^−1^, while the theoretical Raman peaks of the other four PAEs are all shifted approximately 0~20 cm^−1^ relative to the experimental Raman peaks.

From the above results, it can be seen that the theoretical Raman spectra of PAEs still have some differences from the experimental Raman spectra. These differences may be caused by the following reasons. First, the Raman instrument has accuracy problems. Second, the DFT may take the electronic correlation too much into account, leading to calculation errors [20]. Third, theoretical studies generally calculate the structure of individual molecules, while the substances detected experimentally are multimolecular [23]. There are interactions between molecules, and this leads to errors between theory and experiment.

### 3.5. Vibration Mode Assignment of Raman Peaks

The above results show that the theoretical Raman spectra of five PAEs (DMP, DEP, DBP, DEHP, and DINP) calculated by DFT B3LYP 6-311G(d, p) are the best in agreement with the experimental Raman spectra, and have the least spurious peaks. Combining the experimental and theoretical spectra, the Raman peaks of the five PAEs were assigned. Table 3 shows the common Raman peaks and vibrational mode assignments of the five PAEs. Table 4 shows the unique Raman peaks and vibrational mode assignments of the five PAEs. The five PAEs can be identified by their unique Raman peaks.

## 4. Conclusions

In this study, the theoretical Raman spectra of five PAEs (DMP, DEP, DBP, DEHP, and DINP) were calculated using different theoretical methods and basis sets, and the best theoretical method was determined by comparing with the experimental spectra. Also, the common and unique Raman peaks the of five PAEs were identified, and the vibration modes were assigned to these peaks. The results indicate that DFT is more suitable for the theoretical study of PAEs than HF. In the DFT, the B3LYP method is more accurate than the B3PW91 method to calculated the theoretical spectra of PAEs, and 6-311G (d, p) is most suitable for the theoretical study of PAEs among these four basis sets. So, DFT B3LYP 6-311G(d, p) is the most applicable method for the theoretical calculation of the Raman spectra of PAEs, which can reduce the influence of spurious peaks and help to identify the Raman characteristic peaks of PAEs. This will be beneficial for the detection of trace PAEs and the discrimination of PAEs in food products or human blood in the future. Also, the results of this study will help us to establish a Raman fingerprint for PAEs. In the future, further studies must be considered to detect the trace PAEs in food products or human blood by Raman spectroscopy combined with DFT calculation.

## Figures and Tables

**Table 1:** Details of five typical PAEs.

Name	Abbreviations	Chemical Formula	Molecular Weight	Density (g/cm^3^)	Harm
Dimethyl phthalate	DMP	C_10_H_10_O_4_	194.19	1.18	Suppression of the central nervous system
Diethyl phthalate	DEP	C_12_H_14_O_4_	222.24	1.12	Headaches, dizziness, and vomiting
Dibutyl phthalate	DBP	C_16_H_22_O_4_	278.34	1.05	Teratogenic effect on embryos
Di(2-ethyl)hexyl phthalate	DEHP	C_24_H_38_O_4_	390.56	0.99	Carcinogenic to animals
Diisononyl phthalate	DINP	C_26_H_42_O_4_	418.61	0.97	Some effects on reproduction, development, and cancer

PAEs: phthalic acid esters.

**Table 2:** Common Raman peaks in theoretical and experimental Raman spectra of the five PAEs.

Experimental (cm^−1^)	DFT B3LYP 6-311G(d, p) (cm^−1^)				
	DMP	DEP	DBP	DEHP	DINP
400	392	390	356	384	400
650	642	646	646	646	664
1040	1034	1038	1032	1034	1064
1120	1118	1102	1106	1104	1102
1160	1140, 1152	1152	1152	1154	1145
1284	1264	1244, 1268	1242, 1272	1248, 1294	1284, 1320
1450	1440, 1456	1448	1448	1448	1538
1580	1564	1568	1568	1574	1620
1600	1592	1590	1592	1590	1636
1726	1740	1730	1732	1726, 1750	1786, 1800

PAEs: phthalic acid esters; DMP: dimethyl phthalate; DEP: diethyl phthalate; DBP: dibutyl phthalate; DEHP: di(2-ethyl)hexyl phthalate; DINP: diisononyl phthalate.

**Table 3:** Common Raman peaks and vibrational mode assignments of the five PAEs.

PAEs Type	Theoretical (cm^−1^)	Experimental (cm^−1^)	Assignments	Strength
DMP, DEP, DBP, DEHP and DINP	390	400	γ(C-C of the benzene ring)	m
	634	650	β(Benzene)	s
	1020	1040	β(C-H of the benzene ring)	vs
	1104	1120	β(C-C-O)	m
			β(C-H of the benzene ring)	
	1152	1160	β(C-H of the benzene ring)	s
	1264	1284	υ(C-C-O)	m
			γ(C-H)	w
			β(C-H of the benzene ring)	s
	1438	1450	γ(C-H)	m
	1546	1580	β(Benzene)	m
	1570	1600	β(Benzene)	s
	1696	1726	υ(C=O)	s
			γ(C-H)	w

DMP: dimethyl phthalate; DEP: diethyl phthalate; DBP: dibutyl phthalate; DEHP: di(2-ethyl)hexyl phthalate; DINP: diisononyl phthalate;υ: telescopic vibration; β: in-plane bending vibration; γ: out-of-plane bending vibration; δ: deformation vibration; vs: very strong; s: strong; m: medium; w: weak.

**Table 4:** Unique Raman peaks and vibrational mode assignments of the five PAEs.

PAEs Type	Theoretical (cm^−1^)	Experimental (cm^−1^)	Assignments	Strength
DMP	802	818	δ(O=C-O)	w
			γ(C-H of the -CH3)	m
	960	964	υ(O-CH3)	m
			γ(C-H of the benzene ring)	m
DEP	360	352	υ(C-H of -C2H5)	m
	764	784	γ(C-H of -C2H5)	s
	850	848	β(O=C-O)	w
			γ(C-H of -C2H5)	m
DBP	822	810	β(O=C-O)	w
			γ(C-H of -C4H9)	m
	882	842	γ(C-H of -C4H9)	m
	936	940	υ(O-CH3)	s
	958	962		
DEHP	812	834	β(O=C-O)	w
	824	858	β(O=C-O)	m
			γ(C-H of -C2H3(C2H5)C4H9)	m
	874	894	γ(C-H of -C2H3(C2H5)C4H9)	m
	958	956	υ(C-O-C)	m
DINP	822	822	β(O=C-O)	m
	940	900	γ(C-H of -C7H13(CH3)2)	m
	978	960	υ(C-O-C)	m

DMP: dimethyl phthalate; DEP: diethyl phthalate; DBP: dibutyl phthalate; DEHP: di(2-ethyl)hexyl phthalate; DINP: diisononyl phthalate;υ: telescopic vibration; β: in-plane bending vibration; γ: out-of-plane bending vibration; δ: deformation vibration; vs: very strong; s: strong; m: medium; w: weak.

## Data Availability

The data used to support the findings of this study can be made available by the corresponding author upon request.
